# Reported Behavioural Differences between Geldings and Mares Challenge Sex-Driven Stereotypes in Ridden Equine Behaviour

**DOI:** 10.3390/ani10030414

**Published:** 2020-03-02

**Authors:** Anna Aune, Kate Fenner, Bethany Wilson, Elissa Cameron, Andrew McLean, Paul McGreevy

**Affiliations:** 1 Sydney School of Veterinary Science, Faculty of Science, University of Sydney, Sydney, NSW 2006, Australia; kate@kandooequine.com.au (K.F.); bethany.wilson@sydney.edu.au (B.W.); paul.mcgreevy@sydney.edu.au (P.M.); 2 College of Science, University of Canterbury, Christchurch 8140, New Zealand; elissa.cameron@canterbury.ac.nz; 3 Equitation Science International, 3, Wonderland Ave, Tuerong, VIC 3915, Australia; andrewmclean@esi-education.com

**Keywords:** equine, behaviour, sex, welfare, anthropomorphism

## Abstract

**Simple Summary:**

It has been shown that people within the horse industry have preconceived ideas about horse behaviour, temperament and rideability, based solely on the sex of the horse. Such ideas can have welfare implications, if personnel allow bias to affect their interactions with particular horses. Such welfare implications include employment of harsher training methods, and increased horse wastage. The current study explored data on riders’ and trainers’ reports of ridden horse behaviour. Reported sex-related behavioural differences were evaluated based on 1233 responses from the pilot study of the Equine Behaviour and Research Questionnaire (E-BARQ) survey. Results from the study suggest there are some sex-related differences in behaviour between male and female horses; geldings are more likely to chew on rugs and lead ropes when tied, and mares are more likely to move away when being caught in paddock. However, there was no evidence of sex-related differences associated with behaviour when ridden which may warrant further investigation. Findings from this study may be used to educate riders and trainers about the need to regard behaviour and motivation in ridden horses as sex-neutral.

**Abstract:**

Horse trainers and riders may have preconceived ideas of horse temperament based solely on the sex of the horse. A study (n = 1233) of horse enthusiasts (75% of whom had more than 8 years of riding experience) revealed that riders prefer geldings over mares and stallions. While these data may reflect different sex preferences in horses used for sport, they may also reduce the chances of some horses reaching their performance potential. Further, an unfounded sex prejudice is likely to contribute to unconscious bias when perceiving unwanted behaviours, simplistically attributing them to demographic characteristics rather than more complex legacies of training and prior learning. The current study analysed reported sex-related behavioural differences in ridden and non-ridden horses using data from responses to the pilot study of the Equine Behaviour Assessment and Research Questionnaire (E-BARQ) survey. Respondents (n = 1233) reported on the behaviour of their horse using a 151-item questionnaire. Data were searched for responses relating to mares and geldings, and 110 traits with the greatest percentage difference scores between mares and geldings were selected were tested for univariate significance at *p* < 0.2. Multivariable modelling of the effect of sex (mare or gelding) on remaining traits was assessed by ordinal logistic regression, using a cumulative proportional log odds model. Results revealed mares were significantly more likely to move away when being caught compared to geldings (*p* = 0.003). Geldings were significantly more likely to chew on lead ropes when tied (*p* = 0.003) and to chew on rugs (*p* = 0.024). However, despite sex-related differences in these non-ridden behaviours, there was no evidence of any significant sex-related differences in the behaviours of the horses when ridden. This finding suggests that ridden horse behaviour is not sexually dimorphic or that particular horse sports variously favour one sex over another.

## 1. Introduction

Horses are a source of companionship, leisure activities and sport for humans, and therefore their temperament and behaviour directly contribute to their interactions with humans and conspecifics training, breeding and riding. Historically, horses have contributed significantly to human history, initially serving as a source of food, followed by use for transportation, war and agricultural purposes [1,2,3]. With the increased use of machinery in agriculture, a shift from work to sport, leisure and finally companionship has occurred [1,4,5]. Today, horses are bred and trained mainly for sport and recreational purposes, including competition riding and leisure riding [1,6,7]. As partnership styles within the human–horse dyad have evolved, the value of attributes such as social behaviour and temperament have become increasingly important as they influence horses’ merit as riding partners and companions [8,9].

Within professional sport-horse training circles, sex preferences relating to the use of horses for various horse sports are commonly known to exist. For example, mares are widely valued for polo, equally valued in both racing and show-jumping but, to some extent, less valued in dressage and eventing. In the leisure horse world and more particularly in the non-professional equestrian domain, bias against mares is thought to reflect their perceived unreliability [1,2]. Because these preferences are largely anecdotal, further research is warranted in this domain.

Limited research has been published on anthropomorphic application of stereotypes according to the sex of an animal. However, a recent report suggests that many riders and trainers approach the horse-human dyad with preconceived ideas about horse temperament, based solely on the sex of the horse [6]. Historically, sex-related stereotypes have influenced human interactions, and whilst gender equality has significantly improved in many countries, ingrained sex biases still persist in interpersonal relationships [10,11]. This legacy of human society historically devaluing women may be projected from humans onto animals [10,12]. Sex-related stereotypes are common in equestrian contexts, where mares are sometimes perceived by riders and trainers as having inherent temperament traits that are undesirable [6,13]. This is evident throughout the equestrian literature as riders and trainers often report a preference to work with horses of one sex rather than the other [6,8,14]. As described earlier, this may be anecdotally explained by the various equine temperamental requirements of the diverse horse sports. It may also be based, in part, on the personnel’s preconceived idea of sex-related stereotypes, whereby any unwanted behaviour displayed by mares may be categorised as being sex-related rather than being the product of learning history, normal social behaviour, or underlying conditions or aetiologies [13,15].

Practically, preconceived ideas of sex-related differences in horse behaviour may be detrimental to horses. Unfounded preconceptions can have welfare implications as they may lead to an increase in the use of punishment when mares are perceived as *bossy* or *difficult* [6]. Wastage of mares may also increase, with trainers and riders being less willing to work with mares than with geldings and stallions. Racehorses with reportedly poor temperament, for example, are more likely to be sent for slaughter when exiting the industry compared to horses with injuries and poor performance [16]. Misinterpretation of displayed normal social behaviours of horses may also have deleterious consequences to horse welfare. Indeed, depending on the sport, the jurisdiction and the local rules of competition, oestrous behaviour in mares is sometimes regarded as a condition that can be medicated during training or, if declared, competition [17,18,19]. For example, altrenogest may ameliorate oestrus cycling in competition mares, although the effects of long-term treatment are not well known [20].

Free-roaming horses are polygynous, seasonal breeders that, apart from the occasional solitary individuals, form two main group types; harem-type groups (called bands) and bachelor groups [3,21,22]. In bands, stallions play a central part in controlling the movement of the band mares to minimize extra-group fertilization, using specific behaviours (such as snaking their necks at females) known as herding behaviour [3,23,24,25]. Females and offspring in the band respond to male herding behaviour by moving away as a group [24]. However, the social bonds within a band are also an important determinant of band success, with mares forming long-term stable bonds with each other and with stallions [26]. Bachelors are ephemeral groups of non-breeding males, typically young adult stallions with the occasional older stallion [3,23,27]. Bachelors interact and display agonistic and social behaviours such as fighting, playing and allogrooming [27]. Prior to dispersal, colts interact more with each other in social behaviours (including playing and grooming) than fillies, and they show different styles of play, with colts more interactive than fillies [28]. Indeed, colts may prefer the companionship of other colts not least because of shared play styles, including wrestling and nipping, while fillies are more likely to be seen chasing one another [29]. Thus, there are sex differences in behaviour but these are normally seen within a social context and may pertain to entire horses (mares and stallions). This makes it easier to understand the underlying sex differences in mare behaviour, but may make interpretation of geldings more difficult in relation to expected behaviour of free-ranging horses. Geldings may be most similar to pre-dispersal colts or bachelor males rather than mature band stallions, since they are normally castrated prior to full sexual development. 

Although people within the horse industry often have a preferred sex of horse to train and ride, there is currently low inter-study agreement on the effect of personality differences between males and females in questions relating to trainability, anxiety, responsiveness and excitability. Horse temperament can be defined as innate responses by the nervous system, in contrast to behaviours which are complex traits acquired throughout a horses’ life [30]. Personality traits in horses are important because these traits can influence rider’s perception of horses, and their perceived value as a companion [9,31]. There is scant research on the topic of horse personality, with low inter-study agreement on the topic. While one study found geldings to be more trainable than mares with mares being reported as more anxious and panicked than geldings [14], another study found the direct opposite, with geldings scoring higher than mares on ‘anxiety’ [32]. Le Scolan et al. (1997) found no significant differences in reactivity patterns between mares and geldings [33], a finding which was in agreement with recent research by Sackman and Houpt (2019) [30]. The low inter-study agreement on the topic may be attributed to low sample sizes and study designs. It is possible that owners are more tolerant of perceived negative behaviours in mares due to their residual reproductive value as a broodmare. Geldings, having no reproductive potential, may lose value quickly if they become unsuitable for their intended use, as a result of injury or perceived negative behaviours. This may encourage horse handlers to invest more effort into correcting negative behaviours to maintain a gelding’s value.

The current study was designed to explore if there are differences in owner responses to a 151-item pilot questionnaire for the anticipated Equine Behaviour Assessment and Research Questionnaire (E-BARQ) project, depending on whether the focal horse was a mare of a gelding. Respondents were reporting on one subject at a time and not asked to compare horses. Respondents were unaware of the purpose of the study.

## 2. Materials and Methods

This project was approved by the University of Sydney Human Research Ethics Committee (approval number: 2012/656). The E-BARQ pilot questionnaire was developed with the assistance of an international panel of nine experts in the fields of veterinary science, horse training, horse welfare, elite level competition, equestrian coaching, equitation science and equine behaviour. The questionnaire contained 41 demographic questions covering both horse and owner/handler and was then branched into ridden, 268 questions, or non-ridden, 218 questions, sections. At the conclusion of the questionnaire, respondents were invited to leave feedback. The questionnaire was built using REDCap survey software and accessed via a URL link. Respondents could complete the survey in one session or “save and return” if necessary. As E-BARQ was designed as a longitudinal study and a repeated use by each responded, reporting on a focal horse, it was important that respondents reported only on behaviours the horse had exhibited in the past six months. The period in question, together with the focal horse’s name appeared in each question to remind the respondent of the time-frame and which, if they had more than one, horse they were reporting on. The E-BARQ pilot questionnaire was distributed to an audience of horse enthusiasts via Facebook posts and the email lists of Horses and People Magazine, Equitation Science International and Kandoo Equine. Members of the practitioner panel also assisted with the distribution through their own networks. 

### 2.1. Trait Selection

The EBARQ pilot questionnaire was reviewed for questions which characterised a potentially problematic behaviour that had been evaluated on a five-point ordinal scale. One hundred and fifty-one items were selected. These items explore the likelihood of the focal horse:

- Standing unrestrained (or restrained with only a lead-rope and head-collar) for potentially stimulating husbandry procedures (10 questions);
- displaying undesirable behaviours under saddle or preparing for ridden work (28 questions);
- displaying undesirable behaviours while being led (2 questions);
- displaying fear responses to potentially rare stimuli when not under saddle (12 questions) and when under saddle (12 questions);
- displaying particular defensive/aggressive behaviours in response to working or husbandry stimuli (29 questions);
- displaying behaviours indicative of anxiety in response to being taken away from other horses (9 questions);
- displaying behaviours indicative of anxiety in response to being away from home (6 questions);
- displaying stereotypies/problem behaviours when alone, in paddock or in a stall (28 questions); and
- displaying problematic behaviours when being caught and when being transported (15 questions).

The preliminary EBARQ dataset was then searched for responses relating to mares (females over three years of age) and geldings (male horses who had been castrated). Stallions were excluded from the dataset due to low numbers. To avoid biases towards data arising from questions appearing early in the survey, valid responses were limited to those respondents who completed the entire survey. Responses from participants who failed to complete the survey were thus excluded.

This dataset of responses was then collated to identify which questions among the candidate items had a greater than 5% difference in the mean difference scores belonging to mares and belonging to geldings. The absolute value of the mean difference was divided by the larger of the two means. Responses with a greater than 5% difference are shown in Table 1. Following this these responses were then evaluated for a univariate significant difference between mare and gelding scores using a χ^2^ test [34]. Where expected values were low for some scores, a Fisher’s exact test was also performed [34]. Traits with a *p* > 0.2 were considered candidate items for further analysis.

### 2.2. Multivariable Modelling

The effect of horse sex (mare or gelding) was then assessed by ordinal logistic regression through the use of the cumulative proportional log odds model [35]. Each of the traits reporting a *p* < 0.2 in Table 1 above was assessed using ordinal logistic regression. To facilitate diagnostics of the parallel log odds assumption and correction for multiple comparisons (see below) despite each being modelled separately, the same model form and explanatory variables were used for each model. Residual plots were checked for suitability of model fit and the parallel log odds assumption was checked graphically. Each of the explanatory variables was considered of a priori importance and so were forced into the model.

The model was fit to each trait using the “polr” function of the MASS package for r statistical software [34,36].

The form of the full model was

(1) $$logit\left[P\left(Y\leq j\right)\right]=\;\alpha_{j}+x_{i}\beta^{\prime },\;\;\;\;\;\;\;\;\;\;\;\;\;\;\;\;\;\;\;\;\;\;\;\;\;\;\;\;\;\;\;\;\;\;\;\;\;\;\;\;\;\;\;\;j=1,\;2\;,3,\;4$$

where the dependent variable represented one of the 11 traits of interest and each threshold, j, has its own intercept α_j_, with the constraint that α_1_ < α_2_ < α_3_ < α_4_ . *β^’^* is a vector of effects fixed effects related to a vector of explanatory variables (x_i_).

The explanatory variables considered in the model were sex of horse (levels: mare, gelding); horse age (a variable in years); time spent with horse (a count variable of the approximate n of occasions over 15 min spent with the horse over the preceding 6 months); country in which the horse resides (levels: Australia, Canada, Finland, Germany, New Zealand, South Africa, United Kingdom, United States, Other, Not supplied); age group of the respondent (levels: 12–17, 18–24, 25–34, 35–44, 45–54, 55–64, 65–74); coat colour of the horse (Appaloosa, Bay, Black, Brown, Buckskin, Cremello, Dun, Grey, Other, Overo, Palomino, Piebald, Pinto, Skewbald, Tobiano, Tovero); and Breed of the horse (See Table 2). The selected traits were also modelled for interactions (both rider gender-horse sex) and (horse sex-horse age), which returned non-significant *p*-values or *p*-values with low significance that was not significant after multiple comparisons *p*-value correction. The most significant finding for interactions (which would not survive *p*-value correction) related to aggression when with other horses in the arena. At younger ages mares were less aggressive than geldings but mare aggression increased with age, whereas gelding aggression stayed relatively constant with age. This interaction could be explored with a bigger dataset. 

### 2.3. Correction for Multiple Comparison Problem

Because 151 sets of scores were considered in the initial trait selection based on differences in the mare and gelding scores, the *p* values for sex required correction to control the false discovery rate. To report corrected *p* values for the sex effect on the 11 traits of interest, uncorrected *p* values were sought from the remaining 141 questions not deemed of interest and the full set of *p* values were adjusted using the “p.adjust” function, making use of the Benjamimi-Hockberg method for controlling the false discovery rate [37].

Where possible, the full model described above was used (for responses to 112 questions). However, due to the naturally unbalanced design, occasional rank deficiencies arose for the remaining 29 questions. After attempts to resolve the problem by reducing the levels of the breed and coat colour fixed effects as far as five failed, a reduced model which excluded these variables was used to calculate the sex effect *p* values for final 29 questions and complete the set required to control the false discovery rate.

## 3. Results

Analysis of the 11 behavioural traits found mares to be significantly more likely to move away when being caught (*p* = 0.0032), and geldings to be more likely to chew on lead rope when being tied (*p* = 0.0032), and to chew rugs (*p* = 0.024506). Assessment of the effect of horse sex (mare or gelding) was made through ordinal logistic regression using a cumulative proportional log model (see Table 3).

Corrected as they are also for the demographics (gender and age) of the respondents, the country and breed/coat colour effects, the effect of the age of the horse and the number of interactions between the assessor and the horse from this model may also be of interest (see Table 4).

The association with increased levels of interaction between the assessor and the observed horse for the 11 behavioural traits appear in Table 5.

## 4. Discussion

The purpose of this study was to investigate sex-related differences in ridden and unridden horse behaviour as reported by horse owners and trainers through a pilot version of the E-BARQ survey. The results reveal significant behavioural differences between geldings and mares in three non-ridden behavioural traits. Mares are significantly more likely to move away when being caught in paddocks, and geldings are more likely to chew on ropes when tied, and to chew on rugs. The study did not find sexually dimorphic traits during riding between mares and geldings. 

Non-ridden sex-related differences between mares and geldings have been reported more generally in the literature. It is interesting to consider how the three specific behaviours revealed by the current study may be related to previously reported differences and may have evolved for sexual dimorphism. For example, avoidance behaviour in mares may be an aspect of the female equid ethogram, while chewing leads and rugs may be consistent with the male equid ethogram proclivity toward oral behaviours as described later. For the purposes of this discussion, we shall consider geldings to have vestiges of the behavioural traits of stallions and colts (e.g., with an increased tendency to wrestle with one another and occasionally even to mount mares) [38]. We also acknowledge that some geldings may have been only recently gelded at the time of the report and that their behaviour may reflect the behavioural repertoire of colts. 

Free-ranging horses naturally form groups called bands, which typically consist of usually one stallion (but sometimes two or more stallions) and one to several adult mares and their pre-dispersal offspring [3,21,22,23]. The stallions herd the mares of their respective bands, and move them away from other males and bachelors [25,39]. Members of the band typically form established social groups, usually lasting for years, and almost all band mares are mated exclusively by the harem stallion [39,40]. To keep the band together, the stallion exhibits behaviours such as herding, chasing, head-posture threats, and in some instances overt biting [25]. Research suggests that mares that form stable relationships with their band stay with the groups, and tolerate being controlled/directed by the harem stallion. This may help them achieve higher fecundity and greater lifetime reproductive success, as harassment by band stallions is lower in stable groups [25,39]. Thus, mares are more accepting of herding by stallions and will move away from these chasing cues, ultimately contributing to their biological fitness through higher reproductive success. In this sense, mares are primed to respond to chasing cues more than band stallions. Mares moving away when being caught in paddocks may therefore manifest as an analogue of the innate tendency to move away when chased, even when that was not the intention of the handler [41]. 

One may speculate that the reason mares are harder to catch than geldings is either a result of not being caught/worked regularly, or that they are not caught/worked regularly as a result of being hard to catch. Either way, mares being caught/worked less than geldings may potentially be due to preconceived ideas about their behaviours, as research suggests people may be less willing to work with mares than geldings [6,13,15]. Thus mares moving away when approached in paddocks may be blamed on anthropomorphic characteristics of female horses rather than normal social behaviours [13]. Further research could help to substantiate the relative contributions of innate tendencies and handler effects in the aetiology to this undesirable behaviour. 

Most naïve horses react to humans the same way they react to potential predators, in that they move away to avoid physical or psychological pressure [42]. It is also worth noting that horses that succeed in avoiding people who are trying to catch them may be inadvertently rewarded by extended liberty and autonomy, and moving away thus becomes a learned behaviour [43]. Horses negatively reinforced in this way are more likely to move away next time an attempt is made to catch them.

Preconceived ideas on sexually dimorphic traits in ridden mares may also increase the likelihood of more forceful training methods and administration of punishment if they are perceived as less desirable/capable of performing well when trained [44]. If this is the case, mares may even associate their rider/trainer/owner with punishment and thus be less willing to be caught when out in paddock. Free-ranging mares are sensitive to harassment from other horses and try to minimize this harassment [25]. Our study found no sex-related behavioural differences between ridden mares and geldings. This suggests that even if mares are ridden less sympathetically and trained less effectively than geldings, their behaviour under saddle did not reveal any undesirable responses to these interactions.

A limitation to the current study is that we do not know whether the geldings were chewing their own rug (possibly due to frustration) or whether they were chewing each other’s rugs (allogrooming). We can nevertheless explore both of these prospects. Further analysis of a larger set of data may reveal some smaller significant differences between mares and geldings in other traits. The authors caution that any future work should take care to account for the possibility of type 1 errors and also to consider behaviour differences in terms of the clinical significance of the observed effect sizes.

Horses exhibit normal social behaviours such as allogrooming, which is believed to strengthen the bond between group members, and have a calming effect on the recipient [41,45]. Most horses have preferred associates in the herd and they stand together, usually head-to-shoulder or head-to-tail, and groom each other’s neck, mane and rump using their incisor teeth [3]. There is some evidence, however, that males are more active in play and allogrooming than females, particularly during developmental play [28,29,30]. A recent study of equine personality by Sackman and Houpt (2019) found geldings to be more playful and inquisitive [30]. Araba and Crowell-Davis (1994) reported that foals of the same sex associated more with each other, a finding that was particularly evident in colts [28]. In play, colts may be more likely than fillies to use their teeth [28,29]. These reports of males displaying more oral activity and grooming behaviours could explain the current finding that geldings chew more on rugs. In addition, behaviours such as female responses to being rounded-up/chased in open areas, and increased oral activity in males may reflect features of the equine social ethogram.

It is also worth noting that most geldings are castrated in their first year of life [46], which is prior to development of secondary sexual characteristics seen in stallions, such as circling, dancing and head bowing [3]. It is therefore possible geldings remain in a suspended developmental stage, analogous to pre-dispersal foals, as free-living colts normally disperse between 11–15 months (although some stay with their natal band for longer) [3,47]. This could help explain why geldings were found to exhibit more oral activity compared to mares, as young colts in particular demonstrate a submissive chewing face to reduce adult aggression directed at them [27].

The current study reveals that geldings are more likely to chew on lead ropes when tied up. Perhaps they play with ropes as a form of exploratory behaviour. However, beyond play and allogrooming, chewing on objects may be associated with frustration in horses [43]. Given that geldings are considered more reliable, calmer and predictable than mares, riders and handlers may allow biased ideas of male horse behaviour to influence how they manage horses of either sex [6,13]. It is possible that, as a consequence, geldings are being tied-up more frequently than mares and thus experience more frustration. Further research is required to confirm or dispute this possibility. 

It is worth noting that chewing lead ropes when tied, chewing rugs and pushing handlers when offered food all decreased with age whereas moving away when being caught increased with age. These findings may reflect juvenile behaviours that decline with maturity and the effect of learning, for example, that moving away when being caught brings with it the benefit of avoiding ridden work or that pushing handlers when offered food may lead to punishment.

It is clear from this study that while there are some sex-related behavioural differences between mares and geldings, this particular study revealed no indication of sexual dimorphism in ridden horses. However, it is important to acknowledge that the perceived value of male and female horses for different horse sports among professional sport-horse trainers was not evaluated in the current study owing to the likely low representation of that demographic. Horses have not evolved to be ridden any more than humans have evolved to ride, despite strong artificial selection pressure on horses for riding for hundreds of years. Horse-riding has been excluded from the equine social ethogram, and we should not expect horses to relate riding to an activity that manifest as social sexual differences. Horse-riding is not likely to be perceived by horses as sexual; if it were, distinct sex differences would be reported during foundation training. For example, females would differ to geldings in that they would respond either by rejecting or complying when being mounted and ridden, as they do to stallions [42]. The absence of any sexual differences in the behaviour of horses when ridden is consistent with McGreevy et al. (2009) [42]. 

There may be limitations regarding the generalisability of the current findings to the global horse population. It is possible that people who completed the E-BARQ survey may be more engaged with issues relating to horse behaviour than the rest of the horse community. However, due to the large sample size (n = 1233), the results are likely to be representative for the general horse community. The current findings may be used to educate and inform riders and trainers about the need to regard behaviour and motivation in ridden horses as sex-neutral. Increased awareness and understanding of the equid social ethogram is imperative in eliminating pre-conceived ideas of horse behaviour based on the sex of the horse, to ultimately increase horse welfare. As a result, it is acknowledged that the data set may be skewed because these groups may be, to some extent, atypical of the general horse population. 

## 5. Conclusions

Findings from this study suggest there are some sex-related behavioural differences between mares and geldings when they are not being ridden. Mares are more likely to move away when being caught in paddocks, and geldings are more likely to chew on rugs, and chew on lead ropes when tied. No sexually dimorphic behaviour was found in ridden horses although this area merits further investigation. 

## Figures and Tables

**Table 1:** The traits with more than 5% percentage difference scores between mares and geldings, selected among the 151 candidate items reported on in full by respondents (n = 1233). Traits with univariate *p* values < 0.2 on a χ^2^ test (or Fisher’s exact test) were selected for further analysis.

Question	$\bar{{\bar{X}}_{\mathrm{Mare}}-{\bar{X}}_{\mathrm{Gelding}}}$	Percentage Difference	Higher Score	Χ^2^ Statistic	Χ^2^ *p* Value	Fisher Test *p* Value
Chew lead rope when tied	0.331	20.4%	Gelding	33.408	0.000	**0.000**
Chews rugs	0.146	12.2%	Gelding	23.889	0.000	**0.000**
Will stand for facial area tidied with electric clippers (without sedation)	0.342	11.6%	Mare	7.553	0.109	
Move away when being caught	0.202	10.5%	Mare	14.773	0.005	**0.004**
Push handler when offered food	0.166	9.6%	Gelding	9.270	0.055	**0.055**
Hold up one foot when feeding	0.122	8.1%	Gelding	12.076	0.017	**0.014**
Will stand for body to be clipped with electric clippers (without sedation)	0.237	7.6%	Mare	6.537	**0.163**	
Undoes gates	0.111	7.5%	Gelding	5.657	0.226	
Signs of aggression when signalled to canter on the lunge	0.180	6.8%	Gelding	13.844	0.008	**0.007**
Signs of aggression when ridden in arena with other horses	0.167	6.4%	Gelding	30.766	0.000	**0.000**
Pulls back when tied	0.095	5.8%	Mare	5.413	0.248	0.243
Strongly avoided, shied away from or bolted from umbrellas	0.105	5.8%	Gelding	5.220	0.266	0.282
Walk the fence line repeatedly	0.078	5.3%	Mare	5.545	0.236	0.238
Fail to slow when signalled by a rein or lead rope cue	0.095	5.2%	Mare	4.739	0.315	0.310
Vocalising when taken away from other horses	0.149	5.1%	Mare	3.986	0.408	0.408
Head shake when handled or ridden	0.077	5.0%	Mare	7.616	0.107	**0.105**
Strongly avoided, shied away from or bolted from wild animals	0.093	5.0%	Mare	2.474	0.649	0.643
Signs of aggression when lunged or worked in round pen	0.141	5.0%	Gelding	8.045	0.090	**0.100**

**Table 2:** The distribution of breeds of horse reported on in full by respondents (n = 1233) included as a variable in the multivariable model.

Term	Number	Explanation
Andalusian	16	Pedigree Andalusian
Appaloosa	14	Pedigree Appaloosa
Arabian	51	Pedigree Arabian
Arabian Cross (other)	34	Crossbreds with Arabian ancestry and no Thoroughbred ancestry
Australian Stock Horse	20	Pedigree Australian Stock Horse
Connemara Pony	10	Pedigree Connemara Pony
Crossbred(heavy)	18	Not otherwise listed crossbreeds of heavy sized breeds
Crossbred(horse)	106	Not otherwise listed crossbreeds of horse sized breeds
Crossbred(pony)	44	Not otherwise listed crossbreeds of pony sized breeds
Hanoverian	13	Pedigree Hanoverian
Highland Pony	10	Pedigree Highland Pony
Other_Pedigree (pony)	18	Not otherwise listed pedigree horses of pony sized breeds
Other_Pedigree (heavy)	14	Not otherwise listed pedigree horses of heavy sized breeds
Other_pedigree (horse)	87	Not otherwise listed pedigree horses of horse sized breeds
Other_pedigree (unknown)	34	Pedigree horses of unknown breed
Paint	19	Pedigree Paint
Quarter Horse	65	Pedigree Quarter Horse
Quarter Horse cross (other)	40	Crossbreds with Quarter Horse ancestry and no Thoroughbred ancestry
Standardbred	29	Standardbred
Thoroughbredcross (other)	127	Crossbreds with Thoroughbred ancestry and no Quarter Horse or Arabian ancestry
Thoroughbred_×_Arabian	20	Crossbreds with Thoroughbred ancestry and Arabian ancestry
Thoroughbred_× Quarter Horse	16	Crossbreds with Thoroughbred ancestry and Quarter Horse ancestry
Thoroughbred	126	Pedigree Thoroughbred
Welsh Cob	12	Pedigree Welsh Cob
Welsh Pony	12	Pedigree Welsh Pony
Not Supplied	9	

**Table 3:** The uncorrected and corrected sex effect *p* values, as well as the odds ratio for mares (compared to OR = 1 for geldings). Significant differences appear in bold.

Behavioural Trait	Uncorrected *p* Value	Corrected *p* Value	Sex Effect Coefficient	Odds Ratio for Mares	95% Confidence Interval	
Chew lead rope when tied	**0.000**	**0.003**	−0.892	**0.410**	0.269	0.624
Chews rugs	**0.000**	**0.024**	−1.328	**0.265**	0.126	0.558
Will stand for facial area tidied with electric clippers (without sedation)	0.426	0.748	0.180	1.197	0.769	1.864
Move away when being caught	**0.000**	**0.003**	0.688	**1.990**	1.432	2.767
Push handler when offered food	0.071	0.369	−0.316	0.729	0.518	1.027
Hold up one foot when feeding	0.431	0.748	−0.179	0.836	0.536	1.305
Will stand for body to be clipped with electric clippers (without sedation)	0.543	0.828	0.144	1.155	0.726	1.837
Signs of aggression when signalled to canter on the lunge	0.155	0.525	−0.277	0.758	0.518	1.110
Signs of aggression when ridden in arena with other horses	0.105	0.441	−0.321	0.758	0.492	1.069
Head shake when handled or ridden	0.005	0.076	0.534	1.705	1.180	2.465
Signs of aggression when lunged or worked in round pen	0.026	0.215	−0.507	0.602	0.386	0.940

**Table 4:** Influence of age on 11 behavioural traits with at least 5% mean difference scores between mares and geldings and univariate *p* < 0.2. Significant differences appear in bold.

Behavioural Trait	*p* Value	Coefficient	Odds Ratio per Year	95% Confidence Interval	
Chew lead rope when tied	**0.001**	−0.065	0.940	0.900	0.970
Chews rugs	**0.000**	−0.151	0.860	0.800	0.930
Will stand for facial area tidied with electric clippers (without sedation)	0.070	0.039	1.040	1.000	1.090
Move away when being caught	**0.041**	0.032	1.030	1.000	1.060
Push handler when offered food	**0.038**	−0.034	0.970	0.940	1.000
Hold up one foot when feeding	0.844	−0.004	0.996	0.956	1.038
Will stand for body to be clipped with electric clippers (without sedation)	0.070	0.039	1.040	1.000	1.090
Signs of aggression when signalled to canter on the lunge	0.308	−0.018	0.980	0.950	1.020
Signs of aggression when ridden in arena with other horses	0.203	0.024	1.020	0.990	1.060
Head shake when handled or ridden	0.080	−0.031	0.969	0.936	1.004
Signs of aggression when lunged or worked in round pen	0.370	−0.019	0.981	0.941	1.023

**Table 5:** Interactions between assessor and horse for 11 behavioural traits with at least 5% mean difference scores between mares and geldings and univariate *p* < 0.2.

Behavioural Trait	*p* Value	Coefficient	Odds Ratio per Additional Interaction between Assessor and Horse over 6 Months	95% Confidence Interval	
Chew lead rope when tied	0.058	0.122	1.130	0.996	1.283
Chews rugs	0.207	−0.135	0.874	0.709	1.077
Will stand for facial area tidied with electric clippers (without sedation)	0.297	0.076	1.079	0.935	1.246
Move away when being caught	0.626	−0.026	0.974	0.878	1.081
Push handler when offered food	0.064	0.103	1.108	0.994	1.235
Hold up one foot when feeding	0.143	0.109	1.115	0.964	1.290
Will stand for body to be clipped with electric clippers (without sedation)	0.120	−0.119	0.888	0.764	1.032
Signs of aggression when signalled to canter on the lunge	0.131	0.094	1.098	0.975	1.255
Signs of aggression when ridden in arena with other horses	0.118	0.101	1.106	0.975	1.255
Head shake when handled or ridden	0.174	0.083	1.087	0.964	1.225
Signs of aggression when lunged or worked in round pen	0.618	−0.036	0.965	0.839	1.110
